# Editorial: Artificial Polyploidy in Plants

**DOI:** 10.3389/fpls.2020.621849

**Published:** 2020-12-07

**Authors:** Jen-Tsung Chen, Jeremy E. Coate, Geoffrey Meru

**Affiliations:** ^1^Department of Life Sciences, National University of Kaohsiung, Kaohsiung, Taiwan; ^2^Department of Biology, Reed College, Portland, OR, United States; ^3^Horticultural Sciences Department and Tropical Research & Education Center, University of Florida, Gainesville, FL, United States

**Keywords:** antimitotic agents, artificial polyploidy, flow cytometry, plant biotechnology, whole genome duplication

In higher plants, polyploidy plays a significant role in diversification and speciation to drive evolution. Using biotechnology approaches, polyploidy can be induced artificially, chiefly by antimitotic agents, and is a powerful strategy for plant breeding. Duplicating whole sets of chromosomes not only increase copies of existing genes but produces additional alterations of the genome such as epigenetic changes and modulated gene expression that influences a wide range of phenotypes. Consequently, agricultural and horticultural crops with increased ploidy often exhibit anatomical and morphological changes and enhanced biomass, yield, vigor, and stress tolerance, which are potentially valuable for the commercial success of their products. This Research Topic explores current advances in artificial polyploidy and consists of 12 publications that are further divided into three subtopics, as summarized below.

## Development of Novel Protocols for Inducing Polyploidy

The technology for inducing polyploidy is well-established in some plants, however, there is still a large vacancy for the development of novel protocols in induction, evaluation, and screening.

Triploid breeding is a revolutionizing tool in *Populus* breeding that has resulted in improved growth-rate and wood quality for the timber industry, as well-enhanced stress tolerance. However, limited work has been done to improve efficiency of triploid development in *Populus*. In an effort to optimize triploid breeding in *Populus*, Zhou et al. determined that the rate of 2n pollen production was dependent upon the meiotic stage, injection time, and the interaction between the two factors. In the study, the authors report the most effective method for inducing 2n pollen in *Populus*, and conclude that the rate of triploid production is primarily limited by restricted growth of 2n pollen tubes.

Walnuts are economically important trees and their fruits have been part of the human diet for thousands of years. Luo and Chen used fluorescence *in situ* hybridization, an early-fruiting gene fragment-based analysis, and simple sequence repeats analysis to differentiate walnut cultivars from Sichuan Province in China. The authors found Sichuan walnut cultivars have great variation but not sufficient to recognize the cultivars as a new taxon. They proposed the resulting knowledge could be applied in the identification and breeding of Sichuan walnut cultivars. Additionally, such techniques might be applied in the identification of novel genetic variations resulting from induced polyploidy, particularly in tree species.

Currently, there are limited sexual polyploids in the field of floriculture. Zeng et al. evaluated nine cultivars of an orchid, *Cymbidium* Swartz, and found that 2n male gametes occurred in frequencies ranging from 0.15 to 4.03%, and was genotype dependent. They performed seven pairs of crosses between cultivars and obtained five triploids and two tetraploids. The triploid hybrids showed enhanced *in vitro* regeneration capacity, growth, and flowering. The authors concluded that using 2n gametes to produce triploids is reliable for crop improvement in floriculture.

## The Implications of Polyploidy in Plant Breeding

Synthetic polyploidy is crucial to plant breeding, therefore, it is valuable to explore the implications including disease resistance, stress tolerance, and production enhancement, and so on.

In a study of *Hydrangea macrophylla*, Alexander reported that triploids had improved growth, flower size, and larger stoma than related diploids cultivars. The author further studied reproductive biology on interploidy crosses between diploid and triploid cultivars of *H. macrophylla*. Genome size evaluation of the resulting hybrids revealed that the progeny were genetically unstable, and may have broad potential in breeding programs based on their genetic and phenotypic variability.

*Calendula officinalis* L. is a multipurpose plant for horticulture, cosmetics, and medicinal use, with a complex karyotype. Esmaeili et al. estimated genome sizes in nine commercial cultivars and concluded that they were of allotetraploid background. They induced artificial chromosome doubling in three allotetraploid cultivars by treatment with antimitotic agents. The resulting polyploids had enhanced cell volume and leaf size when compared to the original diploids.

Coffee beans are a popular product globally from plants that belong to the *Coffea* genus. Venial et al. induced polyploids from *C. canephora* and *C. arabica* through indirect somatic embryogenesis, and optimized the conditions, including the timing and concentration of the antimitotic agent, colchicine. Using the adopted procedure, they successfully obtained stable polyploids in both *Coffea* species. The authors proposed the protocol might be applied to other shrubbery and woody species for generating novel polyploids.

*Lippia alba* has economic and medicinal value, but currently, there are no reports of polyploidy induction. Julião et al. established a protocol to induce synthetic polyploids using colchicine, and successfully obtained triploid and tetraploids with altered composition of essential oils when compared with the unimproved individuals.

Triploid citrus produces seedless fruits of great economic value, and has been reported to improve tolerance to abiotic stresses, such as cold stress, which is the major impediment to citrus production. Lourkisti et al. reported that triploid citrus was more tolerant to natural chilling temperatures when compared to diploids, parental cultivars, and one diploid clementine tree. They found triploids to have better photosynthetic properties under natural low temperatures and higher contents of E-β-ocimene and linalool that may contribute to enhanced adaptation to cold stress.

Alien chromosome introgression is a valuable chromosome engineering tool for increasing genetic variation in plant breeding programs. Kwiatek et al. found that lines of *Aegilops kotchyi* Boiss obtained through monosomic addition or substitution had significantly improved resistance to leaf rust. The authors propose a similar approach for developing novel triticale varieties with resistance to leaf rust.

The use of synthetic polyploids in commercial production is currently limited, and the underlying mechanisms are poorly understood. A review of synthetic polyploidy by Ruiz et al. provides a brief history of artificial polyploidy in grafted crops, and refines the methodology for inducing polyploidy. The authors point to the potential application of artificial polyploidy in combating climate change in the future.

## Mechanistic Insights into Polyploidy Biology

Based on our knowledge in the field of polyploidy biology, the deep mechanistic insights are still lacking. Hence, the use of advanced technology toward deciphering mechanisms of alterations of growth and development, morphology, yield, metabolism, sterility, and genome structure and function is crucial.

Allopolyploids can be occasionally obtained by crossing distantly related species. The resulting hybrids often exhibit genetic instability and infertility. Park et al. analyzed meiotic chromosome behaviors in pollen mother cells in a synthesized intergeneric allotetraploid, *xBrassicoraphanus*, which is derived from a cross between *Brassica rapa* (2n = 20) and *Raphanus sativus* (2n = 18). According to the findings, the authors suggest that the prevention of non-homologous interactions between the parental chromosomes in *xBrassicoraphanus* is mainly due to structural dissimilarity of chromosomes and the suppression of crossovers between non-homologous chromosomes which is required for correct meiotic progression and gamete formation.

The hypothesis of “hybridization followed by chromosome doubling” suggests that it is possible to develop a viable hybrid zygote by providing each chromosome with a pairing homolog. Tel-Zur et al. attempted to provide evidence supporting this hypothesis by evaluating a series of behaviors in a group of putative hybrids obtained from embryo rescue following controlled crosses of *H. megalanthus* × *H. undatus*. They found that allohexaploids were formed via chromosome doubling events at a very early development stage, consistent with the “hybridization followed by chromosome doubling” hypothesis.

## Conclusions and Perspectives

This Research Topic collects recent advances in the field of Artificial Polyploidy in Plants, and broadly covers critical topics including novel methodologies, plant breeding strategies, and mechanistic insights. We hope that the current era, characterized by rapid development of molecular tools and approaches, such as multi-omics, high-throughput biology, and emerging biotechnology such as CRISPR genome editing will facilitate further unraveling of artificial polyploidy biology, and in turn result in novel mechanistic insights and cultivars for growers.

## Author Contributions

J-TC, JC, and GM drafted the manuscript. All authors revised and approved the final version.

## Conflict of Interest

The authors declare that the research was conducted in the absence of any commercial or financial relationships that could be construed as a potential conflict of interest.

